# Docetaxel-Encapsulating Small-Sized Polymeric Micelles with Higher Permeability and Its Efficacy on the Orthotopic Transplantation Model of Pancreatic Ductal Adenocarcinoma

**DOI:** 10.3390/ijms151223571

**Published:** 2014-12-17

**Authors:** Yunfei Li, Peiran Li, Mingji Jin, Changgao Jiang, Zhonggao Gao

**Affiliations:** 1 State Key Laboratory of Bioactive Substance and Functions of Natural Medicines, Institute of Materia Medica, Chinese Academy of Medical Science & Peking Union Medical College, Beijing 100050, China; E-Mails: mail_liyunfei@163.com (Y.L.); jinmingji@imm.ac.cn (M.J.); 2 Beijing Key Laboratory of Drug Delivery Technology and Novel Formulations, Institute of Materia Medica, Chinese Academy of Medical Science & Peking Union Medical College, Beijing 100050, China; 3 Institute of Medicinal Biotechnology, Chinese Academy of Medical Science & Peking Union Medical College, Beijing 100050, China; 4 Surgical Department, the Affiliated Hospital of Yanbian University, Yanji 133000, China; E-Mail: lipeiran_2009@163.com

**Keywords:** pancreatic ductal adenocarcinoma, small-sized polymeric micelles, docetaxel, permeability, orthotopic transplantation model

## Abstract

Pancreatic ductal adenocarcinoma (PDAC) elicits a dense stromal response that blocks vascular access because of pericyte coverage of vascular fenestrations. In this way, the PDAC stroma contributes to chemotherapy resistance, and the small-sized nanocarrier loaded with platinum has been adopted to address this problem which is not suitable for loading docetaxel (DTX). In the present study, we used the poly(d,l-lactide)-*b*-polyethylene glycol-methoxy (mPEG-*b*-PDLLA) to encapsulate DTX and got a small-sized polymeric micelle (SPM); meanwhile we functionalized the SPM’s surface with TAT peptide (TAT-PM) for a higher permeability. The diameters of both SPM and TAT-PM were in the range of 15–26 nm. *In vitro* experiments demonstrated that TAT-PM inhibited Capan-2 Luc PDAC cells growth more efficiently and induced more apoptosis compared to SPM and Duopafei. The *in vivo* therapeutic efficiencies of SPM and TAT-PM compared to free DTX was investigated on the orthotopic transplantation model of Capan-2 Luc. SPM exerted better therapeutic efficiency than free DTX, however, TAT-PM didn’t outperformed SPM. Overall, these results disclosed that SPM could represent a new therapeutic approach against pancreatic cancer, but its permeability to PDAC was not the only decisive factor.

## 1. Introduction

Exocrine tumors, which occur in the exocrine cells of the pancreas, are the most common form of pancreatic cancer. These tumors account for well over 95% of all pancreatic cancers, and can occur anywhere along the length of the pancreas. Pancreatic ductal adenocarcinoma (PDAC) is the most common type, making up about 90% of all exocrine tumors. Due to delayed diagnosis and aggressiveness of pancreatic cancer, only 10% of patients are eligible for curative treatment and 90% of patients undergo palliative therapies [1].

Chemotherapy may be used either in the palliative setting for metastatic or locally advanced unresectable tumors, or in resectable disease before (neoadjuvant) or after surgery (adjuvant treatment). However, pancreatic cancer is one of the most intrinsically drug resistant tumors and resistance to chemotherapeutic agents is a major cause of treatment failure in pancreatic cancer. Decades of effort have witnessed the failure of many chemotherapeutic regimens and the current standard-of-care therapy, gemcitabine, extends patient survival by only a few weeks [2,3,4]. Impaired drug delivery is one of the major possible mechanisms of chemoresistance. The limitations of clinical chemotherapy have been ascribed primarily to mechanisms that mediate drug resistance at the cellular level [5]. However, substantial evidence suggests that mechanisms that involve the tumor microenvironment also mediate resistance of solid tumors to chemotherapy. Neoplasms of the pancreas tend to have a characteristic vascularization pattern and adenocarcinomas are often hypovascularized as compared to the surrounding tissue [6]. In addition, the tumor-associated stroma (e.g., carcinoma-associated fibroblasts, CAFs) has been implicated as a physical barrier to the delivery of chemotherapy in PDAC and other solid malignancies [5]. Using a genetically engineered mouse model of PDAC that develops extensive stroma, Olive and colleagues showed improved efficacy of gemcitabine when combined with a Hedgehog antagonist [7]. Tumors in this model are poorly perfused, which hampers the delivery and efficacy of gemcitabine treatment. However, treatment with a Hedgehog antagonist depleted tumor-associated stroma and improved tumor vascularity, increased intratumoral concentration of gemcitabine, and stabilized disease [7]. Therefore, there is a dire need for designing new and targeted therapeutic strategies that can overcome the chemoresistance and improve the clinical outcome for patients diagnosed with PDAC.

Advances in nanotechnology have shown great promise in improving the therapeutic efficiency for chemotherapeutic agents in cancer therapy. A variety of nanocarriers and vehicles have been widely studied to transport diagnostic or therapeutic agents to cancer cells [8,9,10]. However, poor penetration of antitumor drugs into the extravascular tumor tissue is often a major factor limiting the efficacy of cancer treatments. The strategy of using small-sized nanocarriers to deliver a chemotherapeutic drug deeply into the tumor has drawn much attention recently [11,12]. H. Cabral *et al.* compare the therapeutic effects of 30, 50, 70 and 100 nm drug-loaded polymeric micelles against PDAC, and found only the 30 nm micelles could penetrate poorly permeable pancreatic tumor to achieve an antitumor effect [13]. Hence, rationally decreasing the size could increase the penetration of nanomedicines, which is potentially to overcome the penetration obstacles against PDAC.

Transportation of agents by nanocarriers depends largely on agent structures [14], and the aforementioned small-sized nanocarrier has been found to be suitable for incorporating platinum agents because of their electrostatic interactions and hydrophobic forces, but has not been shown to be suitable for hydrophobic taxanes (e.g., paclitaxel and docetaxel (DTX)) [13,15]. Taxanes demonstrate a high level of clinical activity, represented by clinical remissions in advanced ovarian, breast and the upper aerodigestive tract cancers [16,17,18]. The central role of taxanes in the therapy of common epithelial cancers is further highlighted by their ability to induce remissions in patients with anthracycline- or *cis*-platinum-resistant epithelial cancers [18]. DTX, in particular, is broadly indicated for the treatment of non-small cell lung cancer, and breast, prostate, stomach and head and neck cancers [19], though these results remain open to debate [20], and is clinically preferred to paclitaxel [19]. However, to our best of our knowledge, very few studies have tested the efficacy of small-sized nanoparticles to deliver DTX in PDAC, and developing a small (10–30 nm) nanocarrier for DTX is desperately needed.

In addition, oncology drug development relies heavily on mouse models bearing transplanted tumors for efficacy testing of novel agents. However, such models of PDAC respond to numerous chemotherapeutic agents, including gemcitabine [21,22,23,24,25,26], suggesting that their predictive utility may be limited. The experimental models used in these studies will never faithfully reflect the clinical disease. Orthotopic human pancreatic cancer xenografts models offer an alternative to transplantation models for preclinical therapeutic evaluation and are used as the preference for cancer research due to the increased clinical relevance and similar to the ideal of the “anthropomorphic” pancreatic cancer model [27].

DTX-loaded small-sized polymeric micelles (SPM) which assemble from amphiphilic block copolymers, poly(d,l-lactide)-*b*-polyethylene glycol-methoxy (mPEG-*b*-PDLLA), have shown great advantages because of the high drug loading capacity, higher permeability and controlled release profiles by our previous study [28,29]. Better penetrating capability would be achieved by modification with TAT peptide [30] at the surface of SPM (TAT-PM). In this study, SPM and TAT-PM were used as the vehicle for DTX against PDAC. The aim of this study was to investigate the therapeutic efficiencies of SPM and TAT-PM in the clinically relevant PDAC (Capan-2 Luc) orthotopic xenografts compared to Duopafei (free DTX; Qilu Pharm. Co., Ltd., Jinan, China). As TAT-PM probably being with elevated permeability, the therapeutic difference would tell us how important the permeability is and provide us an important clue to the future design of small-sized nanoparticles for the PDAC therapy.

## 2. Results

### 2.1. Preparation and Characterization of SPM and TAT-PM

**Figure 1:**
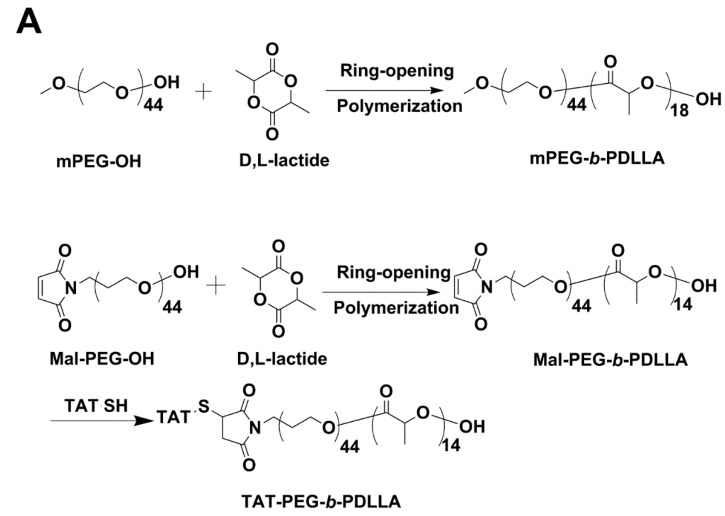

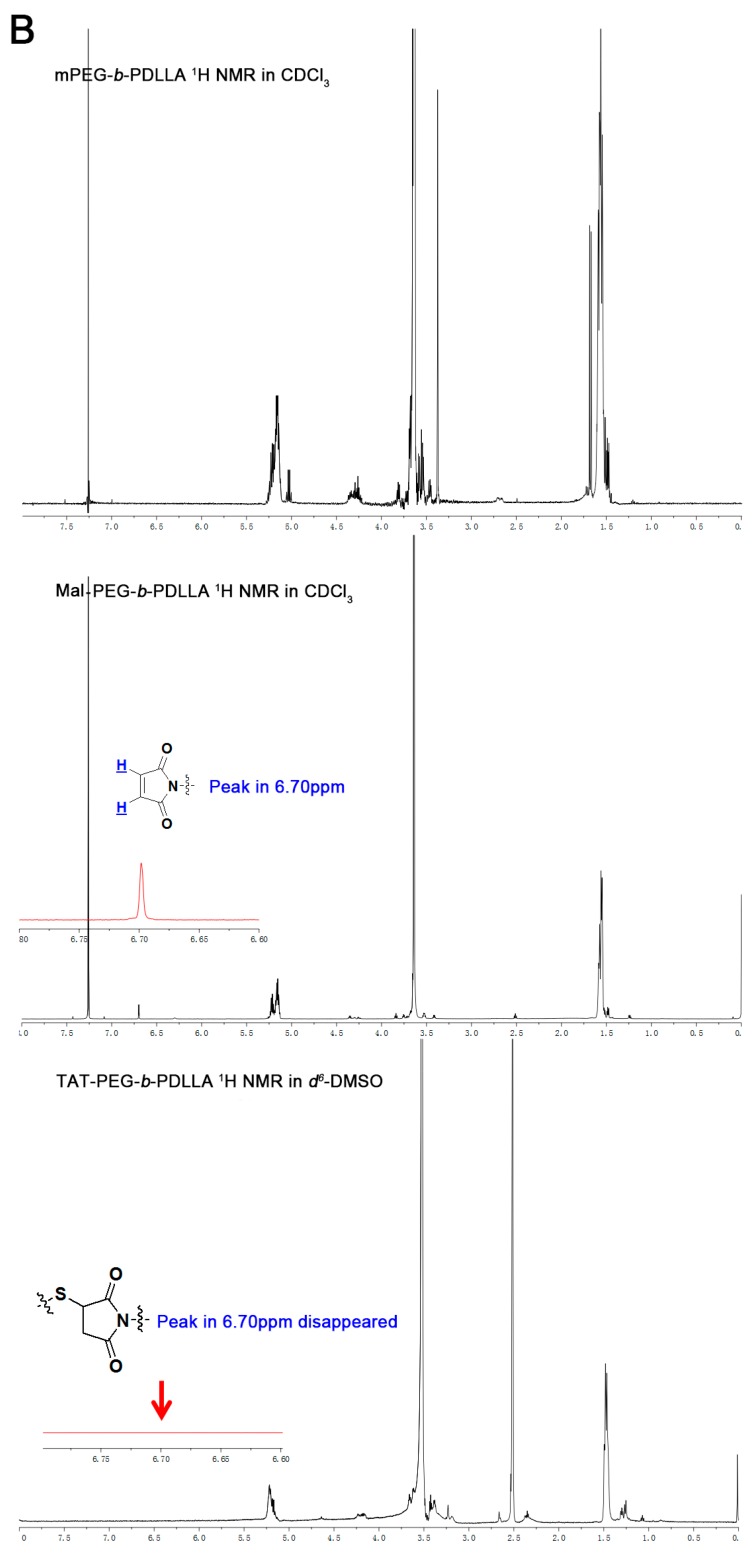

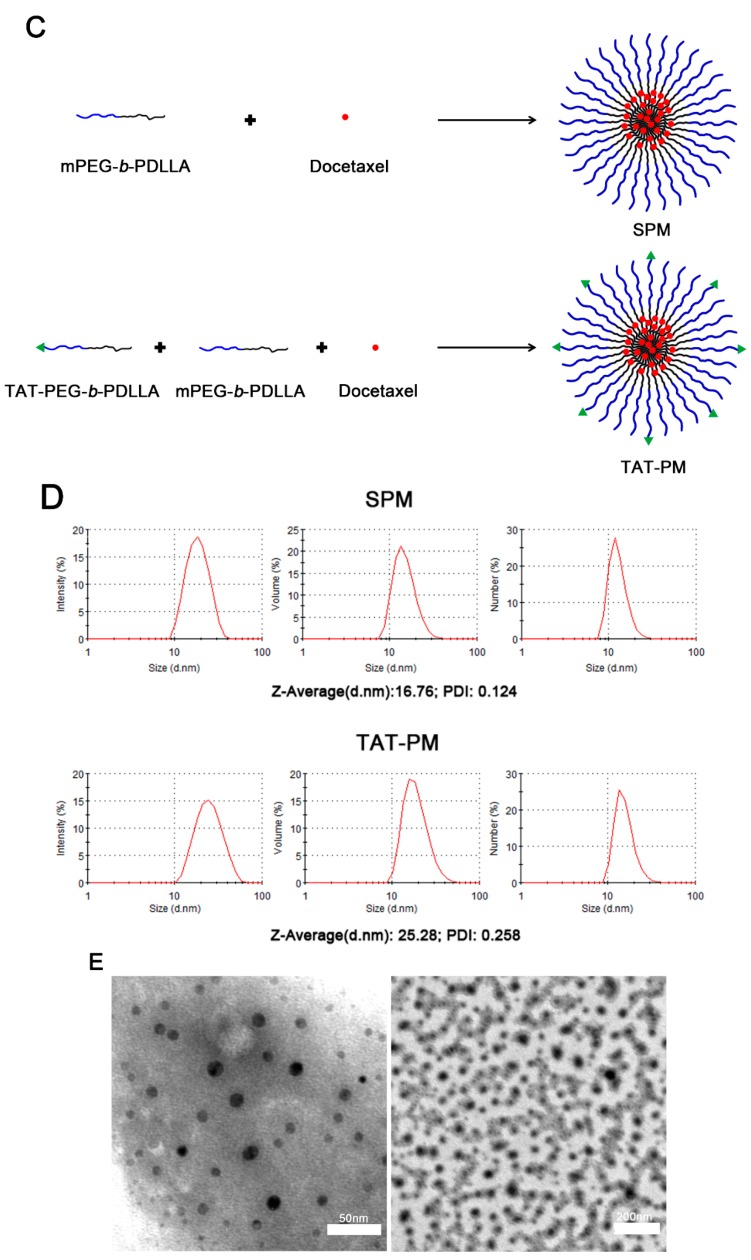

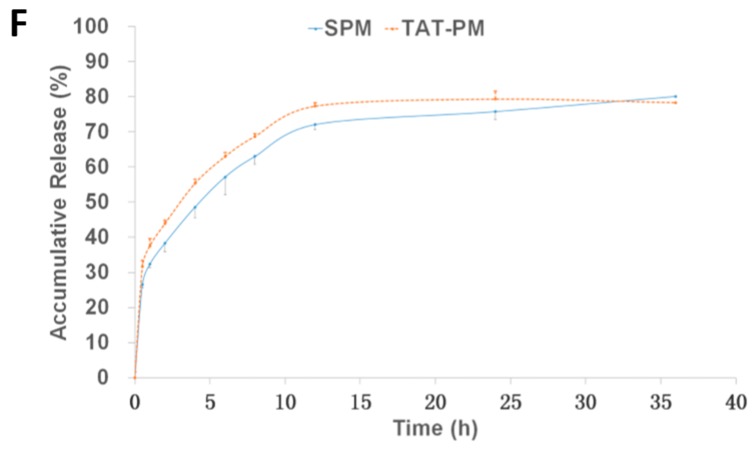
The characterization of small-sized polymeric micelles (SPM) and TAT-PM. (**A**) The synthesis scheme of mPEG-*b*-PDLLA, Mal-PEG-*b*-PDLLA and TAT-PEG-*b*-PDLLA; (**B**) 600 MHz ^1^H NMR spectra of mPEG-*b*-PDLLA, Mal-PEG-*b*-PDLLA and TAT-PEG-*b*-PDLLA; (**C**) Schematic illustration of SPM and TAT-PM; (**D**) Size distribution of SPM and TAT-PM in aqueous medium measured by dynamic light scattering (DLS) analysis; (**E**) Transmission electron microscopy (TEM) images of SPM and TAT-PM; scale bar = 50 and 200 nm respectively; and (**F**) *In vitro* release profile of SPM & TAT-PM in PBS (pH 7.4).

As shown in Figure 1A, mPEG-*b*-PDLLA and Maleimide-PEG-*b*-PDLLA (Mal-PEG-*b*-PDLLA) were synthesized by ring opening polymerization of d,l-lactide [31]. The degree of polymerization of PDLLA was calculated by comparing integral intensity of characteristic resonance of PDLLA at 5.2 ppm (–C(O)–CH(–CH_3_–)) and PEG resonance at 3.64 ppm (–OCH_2_CH_2_–) in the ^1^H NMR spectrum (as shown in Figure 1B). The calculated results indicated that Mn of Mal-PEG-*b*-PDLLA was 3000 Da. Then TAT-SH was conjugated with Mal-PEG-*b*-PDLLA. The excessive TAT-SH was removed by dialysis (MWCO 3500 Da; Millipore, Billerica, MA, USA) against distilled water. The successful conjugation of TAT-SH to Mal-PEG-*b*-PDLLA was further confirmed by ^1^H NMR. As shown in Figure 1B, the absence of the characteristic resonance of maleimide at 6.7 ppm indicated the conjugation as described previously [31]. The CMC of mPEG-*b*-PDLLA was determined as 2.90 µg/mL by a fluorescence spectroscopy measurement in our previous study [28], while the CMC of TAT-PEG-*b*-PDLLA was determined as 5.57 µg/mL using the same measurement method as well.

Then we incorporated DTX into SPM and TAT-PM (shown in Figure 1C) using the self-assembly procedure. Their sizes were examined by dynamic light scattering (DLS). As shown in Figure 1D,E, SPM and TAT-PM had a unimodal size distribution, and the mean diameter were 16.76 and 25.28 nm, respectively. The average micelle sizes of both formulations were in the range of 15–25 nm. The *in vitro* release behavior of SPM and TAT-PM presented as the cumulative percentage release is shown in Figure 1F, which demonstrated that TAT-PM was less stable than SPM probably because of the surface functionalization by TAT peptide.

### 2.2. In Vitro Cytotoxicity Assays

**Figure 2:**
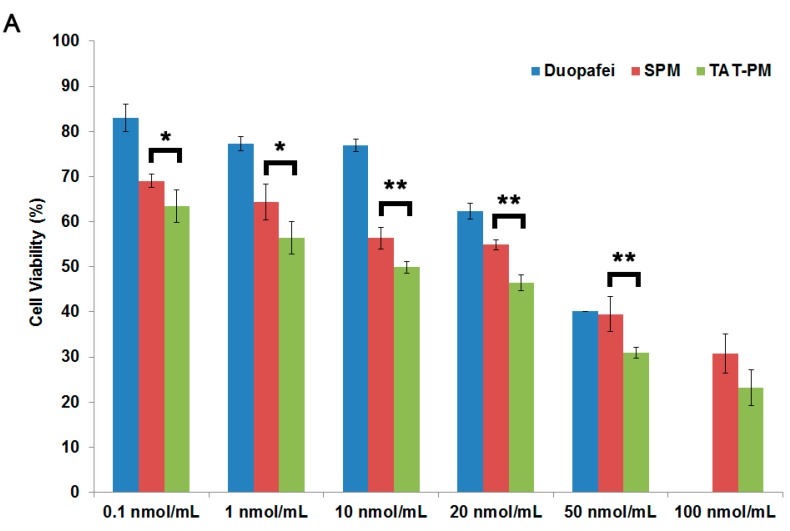

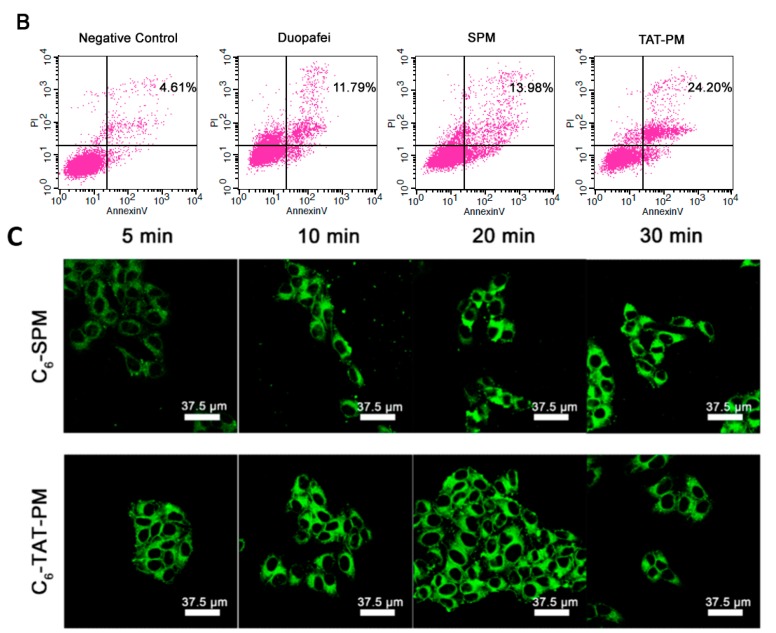
The *in vitro* assessment of SPM and TAT-PM. (**A**) Cytotoxicity effect of Duopafei, SPM and TAT-PM on Capan-2 Luc cells, which was assessed by the MTT assay. SPM treated group *vs.* TAT-PM treated group: * *p* < 0.05, ** *p* < 0.01; (**B**) Confocal laser scanning microscopy (CLSM) images of the Capan-2 Luc cells incubated with SPM and TAT-PM at 37 °C for 5, 10, 20 and 30 min respectively, scale bar = 37.5 μm; and (**C**) Flow cytometry detected cell apoptosis of Capan-2 Luc cells incubated with 10 nmol/mL Duopafei, SPM and TAT-PM for 48 h.

We sought to determine whether encapsulation of DTX in SPM or TAT-functionalized micelles would increase drug entry into tumor cells and cytotoxicity. Capan-2 Luc cells were exposed to a series of equivalent concentrations of Duopafei, SPM and TAT-PM for 48 h, and the percentage of inhibiting rate was quantified using the MTT method. Figure 2A shows the cell viability after 48 h incubation as a function of the DTX amount used for Duopafei, SPM or TAT-PM. Duopafei, SPM and TAT-PM demonstrated the striking dose-dependent cytotoxicities against tumor cells. At the DTX-concentration range of 0.1–50 nmol/mL, SPM and TAT-PM demonstrated higher cytotoxicities than Duopafei against Capan-2 Luc cells as shown in Figure 2A. Especially, there is a significantly higher cytotoxicity with TAT-PM compared to SPM (*p* < 0.05). This could be explained by the increased interaction of TAT-PM with cells because of TAT peptide [30].

### 2.3. SPM and TAT-PM Increased DTX-Induced Apoptosis

DTX has been described as an antimitotic agent that binds to β-tubulin, resulting in block of the cell cycle at the G_2_/M phase and apoptosis of cells [18,19]. Encapsulation of DTX in nanoparticles could induce more apoptosis of prostate cancer cells through the activation of the caspase-2 pathway [32]. Given that SPM and TAT-PM demonstrated stronger *in vitro* cytotoxicity than Duopafei, we performed apoptosis assays using Annexin V-FITC and PI staining to compare apoptosis induction among Duopafei, SPM and TAT-PM. As predicted, SPM increased late apoptosis in Capan-2 Luc cells compared with Duopafei (13.98% *vs.* 11.79%); moreover, TAT-PM induced more late apoptosis than SPM (24.20% *vs.* 13.98%) (Figure 2B).

### 2.4. Interaction to Capan-2 Luc Cells

Confocal microscopy was used to observe internalization speed of SPM and TAT-PM. For the *in vitro* fluorescence imaging investigation, the near-infrared fluorescent probe Coumarin 6 (C_6_) was loaded into mPEG-*b*-PDLLA micelles or TAT-PEG-*b*-PDLLA/mPEG-*b*-PDLLA micelles to yield C_6_-SPM or C_6_-TAT-PM respectively. The CLSM images of Capan-2 Luc cells after incubation with C_6_-SPM and C_6_-TAT-PM for 5, 10, 20 and 30 min are demonstrated in Figure 2C. 5 min CLSM images in Figure 2C showed that the fluorescence of C_6_-SPM in Capan-2 Luc cells was weaker than C_6_-TAT-PM, which indicated that the internalization process of C_6_-SPM was slower than C_6_-TAT-PM. These results give us strong evidence that TAT modified micelles show the significant tumor penetration efficiency to Capan-2 Luc cells compared to plain micelles.

### 2.5. Antitumor Effect of Duopafei, SPM and TAT-PM in Capan-2 Luc Human Pancreatic Cancer Model

**Figure 3:**
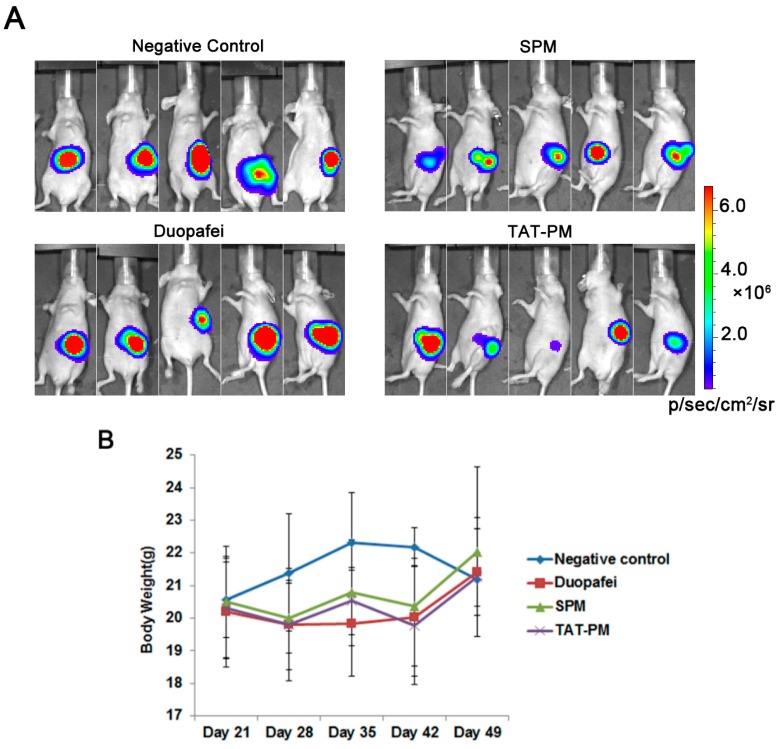

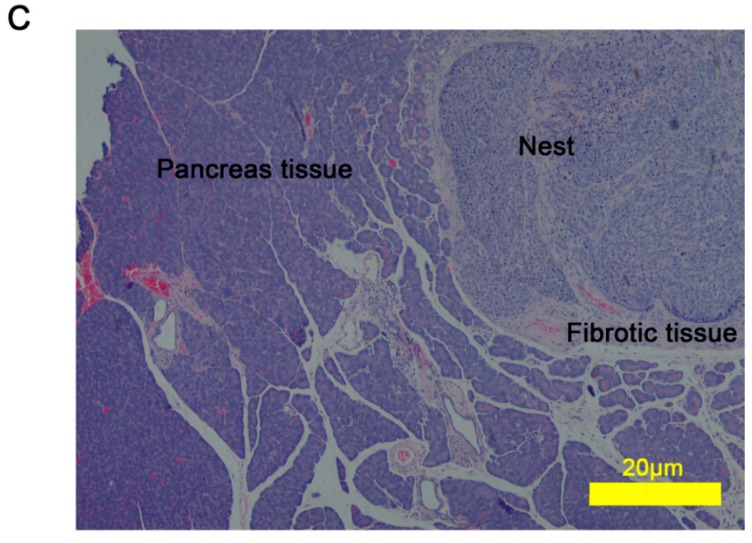
*In vivo* anticancer efficacy of Duopafei, SPM and TAT-PM in the orthotopic transplantation model of Capan-2 Luc. (**A**) The bioluminescent images of nude mice after treated with 5% glucose solution (Negative control), Duopafei, SPM and TAT-PM. Red signal represents the highest level on the colorimetric scale; (**B**) Body weight changes of mice bearing Capan-2 Luc human pancreatic cancer xenografts treated with 5% glucose solution (Negative Control), Duopafei, SPM and TAT-PM; and (**C**) The representative pictures of histopathologic examination of Capan-2 Luc tumor, scale bar = 20 μm.

In order to test the *in vivo* therapeutic efficiency, the treatment by Duopafei, SPM and TAT-PM commenced on day 21 and terminated on day 49 after inoculation of Capan-2 Luc tumor cells. In Figure 3A, the fluorescence of the pancreas *in situ* at day 49, suggested that the transplanted tumor has been well established. SPM achieved a good control of tumor growth, while TAT-PM having the equal therapeutic effects to SPM, as evidenced by the lowest luciferase activity compared with the negative control group. However, Duopafei showed no effect against tumor growth since expressing almost same luciferase activity with negative control group. During the treatment process, animals were weighed at day 21, 28, 35, 42 and 49 (Figure 3B). The body weight changing results indicated that weekly injections were well tolerated with no significant alteration in animal weight relative to saline controls and exhibited no significant difference among the four treatment groups (Figure 3B). Haematoxilyn and eosin (HE) staining of Capan-2 Luc tumors revealed a poorly-vascularized histological pattern and the formation of nests of cancer cells surrounded by fibrotic tissue (Figure 3C), which may act as a barrier against the penetration of drugs and nanocarriers and proved by many studies [13,33,34]. The results showed that SPM and TAT-PM remained high permeability against poorly-vascularized tumors and significantly inhibited tumor growth *in vivo*.

## 3. Discussion

**Figure 4:**
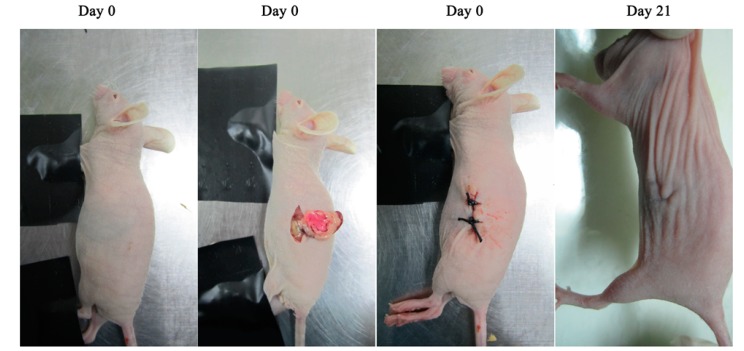
The establishment of the orthotopic transplantation model of Capan-2 Luc.

Despite much effort taken recently, PDAC still presents resistance to currently available conventional treatment approaches [1]. Hence, new drugs discovery to multiple and comprehensive treatments are highly necessary. The appropriate animal models could accelerate the development of therapies and drugs discovery against PDAC. Subcutaneous model could provide visual confirmation that mice used in an experiment have tumors prior to therapy, and provide a means of assessing tumor response or growth over time. However, a major disadvantage of subcutaneous xenografts models is that are curative in mouse subcutaneous xenografts models often do not have a significant effect on human disease. The primary cause of this failure may be due to the observation that the subcutaneous microenvironment is not relevant to that of the organ site of primary or metastatic disease. Orthotopic human pancreatic cancer xenografts models are used as the preference for cancer research due to the increased clinical relevance and similar to the ideal of the “anthropomorphic” pancreatic cancer model [27]. Optical imaging system was also used to detect the real-time growth of Capan-2 Luc human pancreatic tumors. The Capan-2 Luc cell line has been engineered to constitutively express the firefly luciferase gene. As shown in Figure 4, we implanted Capan-2 Luc human pancreatic cancer cells into the pancreas of BALB/c nude mice. When mice carrying Capan-2 Luc tumors are injected with Luciferin the tumors emit a visual light signal that can be monitored using a sensitive optical imaging system like the IVIS Spectrum [35]. The photon flux from the tumor is proportional to the number of light emitting cells and the signal can be measured to monitor tumor growth and development, in this study, we were able to match initial tumor burden between groups, monitor the progression of tumors, and the antitumor activity of micelles in real time by bioluminescence imaging due to the expression of luciferase in the tumors.

The fibrous stroma of PDAC is unusually dense, forming up to 90% of the tumor volume in some patients, and its interactions with the malignant epithelial cells are thought to be an important determinant of the aggressive nature of these cancers [36]. Histological investigations using HE staining also revealed the formation of nests of Capan-2 Luc cancer cells surrounded by fibrotic tissue (Figure 3C), which may act as a barrier against the penetration of drugs and nanocarriers [5,7].

It was recently found that the size of the micelles was critical for achieving deep penetration, particularly in poorly permeable malignancies, such as pancreatic tumors, with 30 nm diameter micelles being capable of passing through the vasculature and interstitium and deeply penetrating inside the tumors [13]. The central role of taxanes in the therapy of common epithelial cancers is further highlighted by their ability to induce remissions in patients with anthracycline- or *cis*-platinum-resistant epithelial cancers [18]. However, in conventional chemotherapy using DTX, the gradients of drug concentration in the tumor may cause the cells far from the vasculature to receive sublethal doses, leading to the development of drug resistance [5]. In contrast, we hypothesized that, by using ultra-small polymeric micelles loaded with DTX, most tumor cells may be exposed to therapeutic concentrations of the drug, improving the scenario of tumor recurrence and resistance to the therapy. Our results confirmed the efficacy of DTX-loaded SPM against an orthotopic pancreatic tumor xenograft as they limited the growth of primary tumors compared to Duopafei (Figure 3A), which suggests that SPM may be novel therapies for the treatment of pancreatic cancer.

There haven’t been any studies concerning on surface-functionalized SPM against PDAC so far, hereby the surface-functionalized SPM was developed. Hypothesizing better DTX treatment efficacy against PDAC tumor, TAT-PM with higher permeability was obtained by modifying the surface of SPM with TAT peptide. *In vitro* experiments demonstrated that TAT-PM inhibited Capan-2 Luc cells growth more efficiently and induced more apoptosis compared to SPM and Duopafei (Figure 2). However, TAT-PM didn’t outperformed SPM in the *in vivo* experiments (Figure 3A), which indicated SPM exerted its therapeutic efficiency not totally relying on permeability. Hence, further study is still needed to find out the exact causes of ultra-small nanoparticles against PDAC except for permeability.

## 4. Materials and Methods

### 4.1. Materials, Cell Line and Animals

All reagents and solvents were used as received without further purification. mPEG with a molecular weight of 2000 Da, d,l-lactide and stannous octoate were purchased from Sigma-Aldrich Chemical Corp. (Shanghai, China); DTX was purchased from Norzer Pharmaceutical Co., Ltd. (Beijing, China); Duopafei was manufactured by Qilu Pharm Co., Ltd. (Jinan, China); HO-PEG-Mal with a molecular weight of 2000 Da was purchased from Jenkem Technology Co., Ltd. (Beijing, China); TAT-SH(Gly-Cys-Gly-Gly-Gly-Tyr-Gly-Arg-Lys-Lys-Arg-Arg-Gln-Arg-Arg-Arg) peptides were purchased from GL Biochem. (Shanghai, China); the 3-(4,5)-dimethylthiazol(-*z*-*y*1)-3,5-*di*-phenytetrazoliumromide (MTT) was obtained from Amresco (Solon, OH, USA). All other reagents used were of analytical grade.

Trypsin, fetal bovine serum (FBS) and RPMI-1640 mediums were purchased from Hyclone (Logan, UT, USA); culture flasks and dishes were from Corning (Corning, NY, USA).The Capan-2 Luc cell line is a pancreatic ductal adenocarcinoma (PDAC) cell line, which was kindly provided by Shenghua Zhang in Institute of Medicinal Biotechnology of Peking Union Medical College. Canpan-2 Luc cells were cultured in RPMI 1640 medium supplemented with 10% FBS and incubated in a humidified atmosphere of 5% CO_2_ and 95% air at 37 °C.

Eight- to ten-week old female BALB/c mice and BALB/c athymic nude mice used for antitumor efficacy studies were purchased from Beijing Vital River Laboratories (Beijing, China). Animals were acclimatized in the holding facility prior to beginning the study, and all animal studies were approved by Peking Union Medical College Council on Animal Care. All surgery was performed under sodium pentobarbital anesthesia, and all efforts were made to minimize suffering.

### 4.2. The Synthesis of mPEG-b-PDLLA and TAT-PEG-b-PDLLA

mPEG-*b*-PDLLA was synthesized exactly according to our previous study [28]. Mal-PEG-*b*-PDLLA was synthesized by ring opening polymerization of d,l-lactide at 110 °C as described previously [31]. Briefly, HO-PEG-Mal was used as a macro-initiator. d,l-lactide was added as a monomer and Stannous octoate was added as a catalyst. After reacting for 4 h, the mixture was allowed to cool down to room temperature. Mal-PEG-*b*-PDLLA was purified by dialysis against distilled water. The degree of polymerization of the PDLLA was determined by ^1^H NMR according to the previous studies [28,31]. For the conjugation of TAT-PEG-*b*-PDLLA, Mal-PEG-*b*-PDLLA was dissolved in acetonitrile; then rotary evaporated to form thin film at 37 °C and hydrated with HEPES solution (pH 8.0). TAT-SH was added and the reaction was kept stirring overnight under N_2_ atmosphere. The excessive TAT-SH was removed by dialysis against distilled water. All the micelle materials were characterized via ^1^H NMR 600 MHz using a Mercury spectrometer (Varian Inc., Palo Alto, CA, USA).

### 4.3. Preparation of Micelles Encapsulating DTX

DTX-loaded Small-sized Polymeric Micelle (SPM), was prepared by thin-film hydration method according to our previous study [28]. The DTX-loaded micelle of TAT-PEG-*b*-PDLLA (TAT-PM) was prepared via co-precitation method as follows. Briefly, TAT-PEG-*b*-PDLLA (1.25 μmol), mPEG-*b*-PDLLA (6.25 μmol) and DTX (6.68 μmol) were mixed in a DMF solution (1 mL), which was then added dropwise to a water solution (10 mL). The final solution was purified by dialysis (membrane MWCO 12,000–14,000) to give TAT-PM.

Both DTX-loaded micelles were extruded through a membrane of poresize 220 nm. The sample was diluted with water to yield 1 mg/mL final DTX concentration, as determined by high-performance liquid chromatography (HPLC, 1200 series; Agilent Technologies, Palo Alto, CA, USA) with a pentafluorophenyl column (Curosil-PFP, 4.6 mm × 250 mm, 5 μm; Phenomenex, Torrance, CA, USA). Size distribution of the micelles was investigated by dynamic light scattering (DLS) method using Nano ZS90 (Malvern Instruments Inc., Worcs, UK).

The release profile of DTX from SPM and TAT-PM was evaluated using a dialysis membrane method: 0.5 mL of SPM or TAT-PM solution at a DTX concentration of 1 mg/mL was placed in a dialysis bag (MWCO 3500). The dialysis bag was incubated in 40 mL of phosphate buffered saline (PBS, pH 7.4) at 37 °C with gentle shaking at 100 rpm, and aliquots of incubation medium were removed at predetermined time points. DTX in the samples was quantified by HPLC using the above method.

### 4.4. In Vitro Cytotoxicity

For *in vitro* cytotoxicity study, MTT assay was used. Briefly,Capan-2 Luc cells were harvested from exponential phase cultures growing in RPMI 1640 medium supplemented with 10% FBS, counted and plated in 96-well flat-bottomed microtiter plates (5 × 10^3^ cells per well). After a 24 h incubation, cells were then treated with a series of doses of Duopafei, SPM, or TAT-PM, respectively. After 48 h of incubation, 20 μL of MTT solution (5 mg/mL) was added to each well of the plate. After incubating for additional 4 h, MTT was aspirated off and 200 μL/well of DMSO was added to dissolve the formazan crystals. Absorbance was measured at 490 nm by a microplate reader (Synergy H_1_/H_1_ MF; Bio-Tek Inc., Winooski, VT, USA). Untreated cells were taken as control with 100% viability and cells without addition of MTT were used as blank to calibrate the spectrophotometer to zero absorbance. The results were expressed as mean values ± standard deviation of five measurements.

### 4.5. Cell Apoptosis Assay

Apoptotic cells were determined by dual staining with an Annexin V and propidium iodide (PI) kit (China KeyGEN Biosciences Company, Nanjing, China) according to the manufacturer’s instructions. After 48 h of culture in the exponential stage, Capan-2 Luc cells seeded in 12-wellplates were treated for a further 48 h with 10 nmol/mL Duopafei, SPM or TAT-PM, respectively. After treatment, cells were washed twice with warm PBS, detached by trypsin without EDTA, collected, centrifuged, washed with warm PBS, resuspended in the binding buffer and further stained with PI and Annexin V-FITC for 15 min at ambient temperature in the dark. Apoptosis was then analyzed using a FACScan cytometer equipped with Cell Quest software (BD Biosciences, San Jose, CA, USA). Quadrant analysis was performed and cells that stained positive for both Annexin V-FITC and PI were designated as apoptotic, while unstained cells were designated as live.

### 4.6. Cellular Uptake of C_6_-SPM or C_6_-TAT-PM

For the *in vitro* fluorescence imaging investigation, the near-infrared fluorescent probe C_6_ was loaded into mPEG-*b*-PDLLA micelles or TAT-PEG-*b*-PDLLA/mPEG-*b*-PDLLA micelles to yield C_6_-SPM or C_6_-TAT-PM respectively. In brief, the polymers and C_6_ were co-dissolved in CHCl_3_ and a thin film formed by evaporation of CHCl_3_. Phosphate Buffered Solution (PBS, pH 7.4) was added, followed by votex for 10 min. The micelle suspension was extruded through a membrane of poresize 220 nm to remove the free C_6_.

Capan-2 Luc cells were cultured in RPMI 1640 medium supplemented with 10% FBS. The culture was maintained in incubator containing 5% CO_2_ at 37 °C. The cells were incubated with C_6_-SPM and C_6_-TAT-PM at 37 °C for 5, 10, 20 and 30 min, rinsed with cold PBS for three times and then fixed by 4% paraformaldehyde for 10 min. Finally, cells were observed by confocal laser scanning microscope (CLSM, TCS SP2; Leica, Wetzlar, Germany). The images of the cells were determined with differential interference contrast (DIC) channel, and the images of C_6_-SPM and C_6_-TAT-PM were recorded with green channel (C_6_) with excitation at 488 nm.

### 4.7. Capan-2 Luc Orthotopic Transplantation Human Pancreatic Tumor Model

Capan-2 Luc cells growing in exponential phase were diluted to 1.5 × 10^7^ cells/mL in exponential stage growth with RPMI 1640 medium. Nude mice were anesthetized with injection of 60 mg/mL pentobarbital sodium intravenously, and followed by making a small incision into the abdomen along the lower left rib cage to pull out the pancreas and inject 3 × 10^5^/0.02 mL of Capan-2 Luc cell suspension into it. Incision suture was operated according to conventional surgery. The whole surgery process was demonstrated in Figure 4. On day 21 after inoculation, the mice were randomized into 4 equal groups (*n* = 5 for each group) for treatment: negative control (5% glucose solution, 0.2 mL/mice), Duopafei, SPM and TAT-PM. Each formulation was injected intravenously via the tail vein at a dose of 10 mg DTX/kg body weigh every 7 days for 21 days and the mice body weights were measured simultaneously. At day 49 after inoculation, luciferase substrate d-luciferin (150 mg/kg) was injected intraperitoneally, and the mice were placed onto the warmed stage inside the camera box (IVIS-live Imaging System 200; Xenogen Corp., Alameda, CA, USA) to observe tumor growth. Then all mice were sacrificed, and representative pancreatic tissues were taken for HE staining after formalin-fixation and paraffin-embedding.

### 4.8. Statistics

Data were described as means ± SD of the indicated number of individual experiments. If there was significant variation between treatment and control groups, the mean values were compared using Student’s *t*-test. *p*-values less than 0.05 were considered statistically significant difference and *p*-values less than 0.01 were considered statistically very significant difference.

## 5. Conclusions

Our findings strengthen the usefulness of polymeric micelles, particularly of SPM, for the clinical setting. Delay of disease progression without negatively impacting quality of life may be significant benefits from SPM. And what’s more, we found there should be other cause except permeability for SPM exerting its therapeutic ability against PDAC. Despite considerable progress in demonstrating therapeutic effects of SPM against PDAC, the molecular basis and *in vivo* distribution process underlying its inhibitory functions remains obscure and further experiments are needed to address this issue.
